# One Health training needs for Senegalese professionals to manage emerging public health threats

**DOI:** 10.1016/j.soh.2022.100005

**Published:** 2022-11-26

**Authors:** Walter Ossebi, Andrée Prisca Ndjoug Ndour, Sara Danièle Dieng, André Pouwedeou Bedekelabou, Miguiri Kalandi, Fatimata Niang Diop, Rianatou Bada Alambedji, Yalacé Yamba Kaboret, Adama Faye, Bienvenu Sambou

**Affiliations:** a Inter-State School of Veterinary Sciences and Medicine, Cheikh Anta Diop University, Dakar-Fann BP, 5077, Senegal; b Ministry of Livestock, Togo; c Inter-State School of Veterinary Sciences and Medicine, Africa One Health University Network, Dakar-Fann BP, 5077, Senegal; d Institute of Environmental Sciences, Cheikh Anta Diop University, Dakar-Fann BP, 5005, Senegal; e Institute of Health and Development, Cheikh Anta Diop University, Dakar-Fann BP, 5005, Senegal

**Keywords:** Training needs, One Health, Professionals, Competencies, Senegal

## Abstract

Global health trends, marked by increasing epidemics and pandemics, call for enhanced human resource preparedness to prevent and respond to emerging and future health problems. Indeed, according to the World Organization for Animal Health (WOAH), at least 75% of the pathogens responsible for emerging and re-emerging human infectious diseases originate from animals. These health problems involve environmental, economic and social factors. Because of their multidimensional nature, addressing these health problems requires the adoption of a One Health approach. To achieve this, training of human resources is a priority.

This descriptive cross-sectional study was conducted from September 12 to 22, 2021 in 6 regions of Senegal, namely, Dakar, Thiès, Diourbel, Kaolack, Saint-Louis, and Ziguinchor. Questionnaires were administered online and in person to 217 professionals in human, environmental, animal health and agriculture. Data were statistically processed, and bivariate analyses enabled a better correlation between training needs for professionals according to their occupational group.

The survey sample was 64% male and 36% female. More than half of the professionals surveyed (53.5%) had less than 10 years of experience. Human health workers were the most represented (46%), followed by animal health workers (34%) and environmental workers (16%). Agriculture, fisheries, and food security were weakly represented. Few had received training in the One Health approach and entry-level One Health skills predominated. The One Health competencies in which professionals want to build capacity differ by sector. Globally, public health and epidemiology, health risk management, basic of One Health concepts, animal health and biotechnology are the priorities.

The development of training programs could then enable these expressed needs to be addressed.

## Highlights

• More than half of the surveyed professionals had not received training in One Health
• Entry-level competencies in One Health are prevalent among professionals
• Professionals' needs are focused on exploring and learning the One Health approach
• Professionals focus more on technical competencies in “One Health” than on “soft skills
• Professionals focus on competencies related to health risk management, public health and epidemiology

## Graphical abstract

**Figure:**
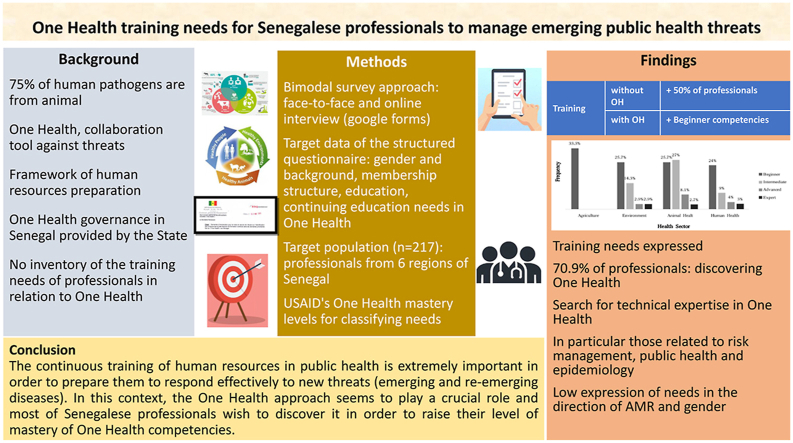

## 1 Introduction

The world is increasingly facing health problems with multiple implications and challenges. According to the World Organization for Animal Health (WOAH), at least 75% of emerging infectious diseases in humans are caused by animal pathogens [1]. Avian influenza, HIV, and Ebola are examples of diseases with a high negative impact on global health. Several factors favour the emergence of diseases, such as climate change, the degradation of natural ecosystems, and a high level of international mobility, which facilitates contact between pathogens and humans. Global health issues continue to become more complex, so countries should train health personnel to cope with them. Yet, global health trends call for increased human resource preparedness to prevent and respond to multiple current and future health challenges. Therefore, the One Health approach offers an opportunity to better prepare these human resources. One Health is defined as “*an integrated, unifying approach that aims to sustainably balance and optimize the health of people, animals and ecosystems. It recognizes that the health of humans, domestic and wild animals, plants, and the wider environment (including ecosystems) are closely linked and interdependent. The approach mobilizes multiple sectors, disciplines and communities at varying levels of society to work together to foster well-being and tackle threats to health and ecosystems, while addressing the collective need for clean water, energy and air, safe and nutritious food, taking action on climate change, and contributing to sustainable development” (*the One Health High Level Expert Panel (OHHLEP).^1^

Senegal, unlike other countries, has an appropriate health workforce. On a scale of 1–5 of the Joint External Evaluation (JEE) of the International Health Regulations (IHR), the workforce development indicator of the country scores lies between 2 and 4 [2]. Senegal's low score (2) on the workforce strategy is related to the lack of a comprehensive national plan for health human resources despite the availability of multidisciplinary resources at national and intermediate levels. Existing public health plans exclude professionals such as epidemiologists, veterinarians and others. Thus, the report recommends that human resource development plans to be developed and implemented in all public health-related sectors, with consideration of IHR and PVS (Performance of Veterinary Service) aspects. Additionally, this report highlights the need for the personnel to be trained in the One Health approach.

The One Health approach is generally accepted and wanted in most countries. Still, the bottleneck is often figuring out how to adjust existing systems and habits to make them practically operational in the relevant sectors. Implementing collaboration takes time and energy, as demonstrated with the IHR and PVS [2,9], the One Health efforts will support early-stage sector-specific goals and mandates with the alignment of ongoing activities and more effective use of limited resources as an added value. The aim is to provide a robust and proven methodology that creates an enabling environment for national staff to identify and discuss their own needs (without relying on standards or a universal progress scale) and derive tailored solutions, adapted to the country's structure and challenges [3]. In Senegal, a One Health consultation and governance framework (called the National High Council for Global Health Security-One Health) has been set up at the Prime Minister's Office for better multisectoral coordination. This framework is a place for co-learning and co-construction of One Health operationalization methods by the actors in implementing it. A shortcoming of this framework, noted by Kabkia et al. [4], is the lack of information sharing on disease surveillance, particularly those with acute epidemic potential. WHO/CDC and FAO have developed training in field epidemiology to fill this gap (Field Epidemiology Training Program [5] and In-Service Applied Veterinary Epidemiology Training [6]) through capacity building of human, animal, and environmental health professionals. However, the training needs of these professionals in relation to One Health, known elsewhere, have not yet been assessed.

This pioneering study aims at identifying and analyzing training needs in One Health for healthcare personnel in Senegal. Specifically, it aims to: (i) describe the sectors of activity (central and peripheral) professionals, (ii) describe their level of study, and (iii) analyze continuing education needs in One Health among health professionals (human, animal, environmental, agricultural, fisheries and aquaculture) at the national level.

## 2 Materials and methods

This descriptive cross-sectional study was conducted from September 12 to 22, 2021 in 6 regions of Senegal, including Dakar, Thies, Diourbel, Kaolack, Saint-Louis and Ziguinchor. These regions host 65% of the human health institutions, and 77% of the health personnel, wildlife reserves (Bandia, Kalissaye, Gueumbeul, etc.), protected forests (Pané, Saboya, Mamby, Mpal, Kalounayes, etc.), and parks (Madeleine Islands National Park, Djoudj National Bird Park, Lower Casamance National Park, etc.), the Chain of Command for human, veterinary and environmental health services [[7], [8], [9]]. The principal targets of this study were human health, environmental, agricultural, and fisheries actors, working primarily in the public sector.

Based on an estimated size of 14,253 health professionals in Senegal in 2016 [7], and the Thrusfield formula [10], a sample size of 200 interviewees was determined for a 5% risk of error, 6.9% accuracy, and 50% proportion. The proportional distribution of this number gave 90, 30, 25, 20, 20, and 15 people to be interviewed in the regions of Dakar, Thiès, Diourbel, Saint-Louis, Ziguinchor and Kaolack, respectively.

A structured questionnaire integrating One Health competencies [11,12] was used for data collection [13]. The questionnaire was subdivided into four main sections: (i) the gender and origin of the interviewee, (ii) the structures to which they belong, (iii) the level of education and (iv) the expressions of the need for continuing education in One Health. The questionnaire was also posted on *Google forms* to reduce contact between individuals due to the sanitation context of COVID-19.

The survey was conducted in two ways: an indirect online interview via Google forms and a face-to-face interview. In a face-to-face mode, health professionals were interviewed using the questionnaire in their workplace. Additional information was requested over the telephone in case needed, and for both face-to-face and online respondents.

Interviewers consisting of physicians, veterinarians and agricultural officers were recruited and trained to cover the different study sites.

### 2.1 Ethical consideration

Ethical approval was obtained from the ethics committee of Cheikh Anta Diop University (UCAD) in Dakar (n°CER/UCAD/ADIMsN/o56/2O2L of 23/08/2021) before the survey began. Similarly, formal informed consent was obtained beforehand from each person to be surveyed.

### 2.2 Data processing and analysis

Collected data on survey forms were saved on Google forms and then exported to Microsoft Excel 365 and Sphinx version 5 for processing.

The Unesco classification [14] was used to rank the responses on an educational level. Similarly, the “One Health” mastery levels defined by USAID [15] were used to assess the level of knowledge of health professionals on this approach (Table 1).

**Table 1:** Mastery levels of the One Health approach.

Level	Description
Biginner/novice	Limited field experience; developing On-the-job experience; understanding of One Health terminology, concepts and principles
Intermediate	Start with practical application; widening knowledge and skills, help required from expert
Advanced	Applied theory; broad professional issues; perform skills without assistance
Expert	Recognized authority; Assessment and supervision, control, strategy, consistency excellence/mastery

Source: USAID, 2020.

R software version 4.1.2 was used for statistical analysis. Descriptive statistics (means, frequencies, standard deviations) and bivariate analyses were first performed on the data to assess the correlation of training needs for professionals according to their occupational group.

## 3 Results

### 3.1 Distribution of surveyed actors

**Fig. 1:**
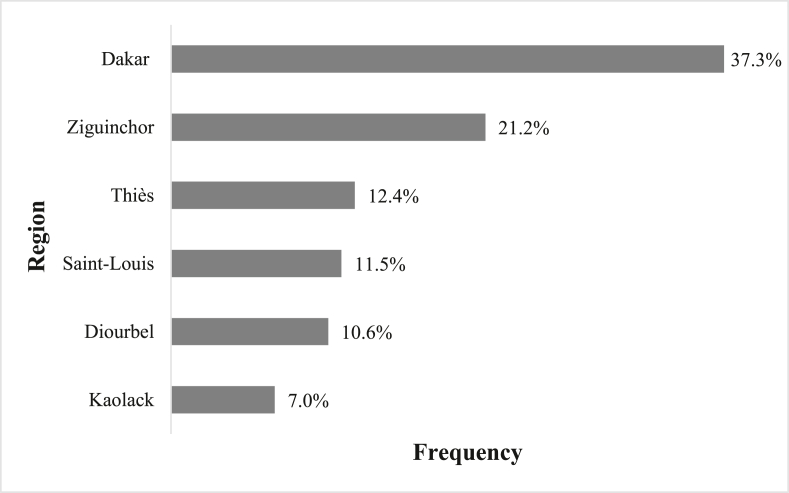
Distribution of interviewees by region.

A total of 217 health actors participated in the survey. The sex gender is not balanced as 64% were men and 36% women. The largest number of respondents (Fig. 1) were in Dakar (37.3%) and Ziguinchor (21.2%). Respondents with more than 10 years of experience (45.2%) in their field were less numerous than those with less than 10 years (53.5%). Slightly less than 2% (1.4%) did not answer this question.

### 3.2 Distribution of professionals according to the sector of activity

**Fig. 2:**
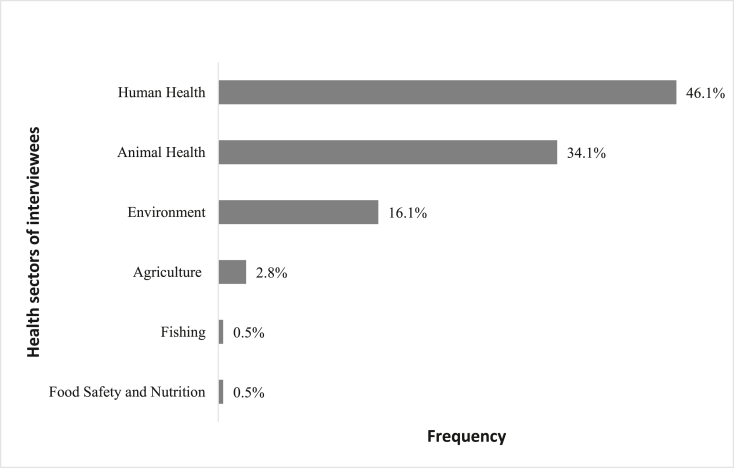
Interviewees' sectors of activity.

Most professionals interviewed (77%) were from the public sector and few from the private sector (17.9%). However, 5.1% interviewees did not answer the question. The field activity's diversity of respondents fits quite well the multidisciplinary aspect requirement of the One Health concept (Fig. 2).

### 3.3 Human health professionals

**Fig. 3:**
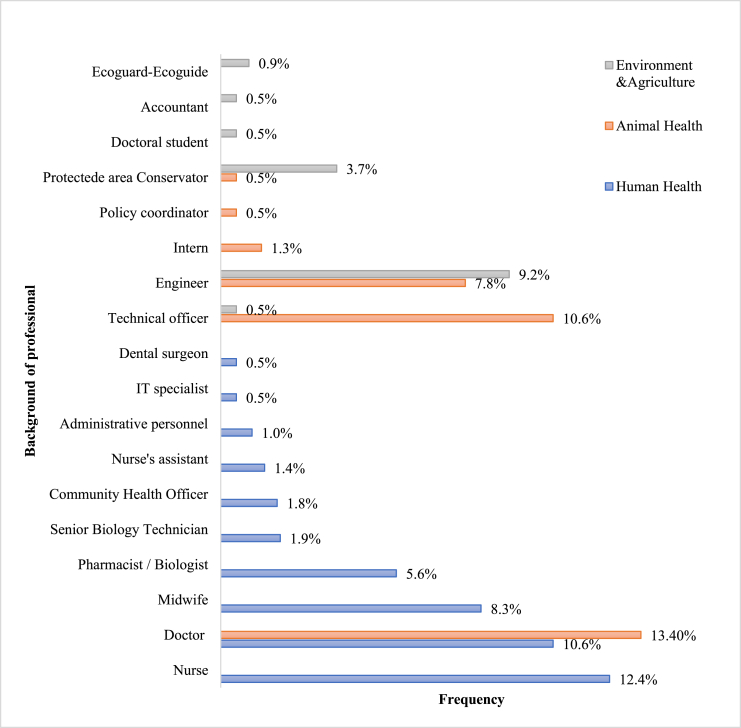
Typologies of professions of interviewees.

Among human health professionals (Fig. 3), nurses (12.4%), physicians (10.6%) and midwives (8.3%) were more common. Except for 6 pharmacists working in the private sector, all human health professionals worked in the public sector, mainly in health centers and hospitals. The remaining professionals work in private clinics, training and research institutions, and national agencies.

### 3.4 Animal health professionals

Veterinary doctors and animal husbandry technicians (ATEs) were the largest group of animal health professionals to participate in the survey (Fig. 3). Almost half (45.2%) of the professionals in this sector work in the private sector.

### 3.5 Agriculture and environment professionals

Only two respondents from the agricultural sector participated in the surveys, while the rest of the stakeholders were from the environmental sector (Fig. 3). Water and forestry engineers, conservationists and agronomists were the most common profiles encountered.

### 3.6 Education level of professionals

The surveyed professionals have educational levels ranging from high school to doctorate (Table 2). Non-university levels are few and are mainly found in the environmental and human health sectors. The secondary level is found among environmental professionals and concerns ecoguides or ecoguards. These are support staff who work in parks and reserves as volunteers and are paid through tourist visits. Post-secondary non-tertiary education is observed among human health professionals, including nursing assistants, who are recruited without baccalaureate. The above-mentioned categories have sometimes not received basic training specific to the fields in which they practice. Their skills are developed either through on-the-job training or through continuing education via public and private projects and institutions.

**Table 2:** Educational level of professionals in Senegal.

Instruction Level	Health Sector			
	Human	Animal	Environment	Agriculture/Fishing
3			Ecoguide/Ecoguard	
4	Nurse Assistant			
5		Livestock Technical officer	Livestock technical officer, Water and Forest Technician,	
6	Nurse, Midwife	Livestock engineer	Water and Forestry engineers, National Park engineer	Agronomist
7	Senior Biologist Technician, Community Health Officer, Administrative personnel, IT scientist		Water and Forestry Inspectors	Agronomist
8	Medical doctor, Dental Surgeon, Pharmacist, biologist	Veterinarian/Conservator	National Park Conservators, Other^a^	

Legends according to the Unesco classification [14].

3-secondary education.

4- post-secondary non-tertiary education.

5-short-cycle higher education.

6-bachelor's degree or equivalent.

7-master's level or equivalent.

8-doctorate level.

a Researchers.

The upper level, which concerns the decision-making levels, is dominant in the training profiles ranging from the short cycle to the long cycle. The short cycle is equivalent to a senior technical certificate and concerns agents working in animal husbandry and water and forestry. The Baccalaureate level is for nurses, midwives, and engineers in livestock farming, water and forestry, and national parks. The master's level refers to laboratory technicians, community and administrative agents, computer scientists and water and forestry inspectors. The doctorate level or equivalent refers to doctors, pharmacists, veterinarians, curators, etc.

### 3.7 One Health competencies needed by professionals

**Fig. 4:**
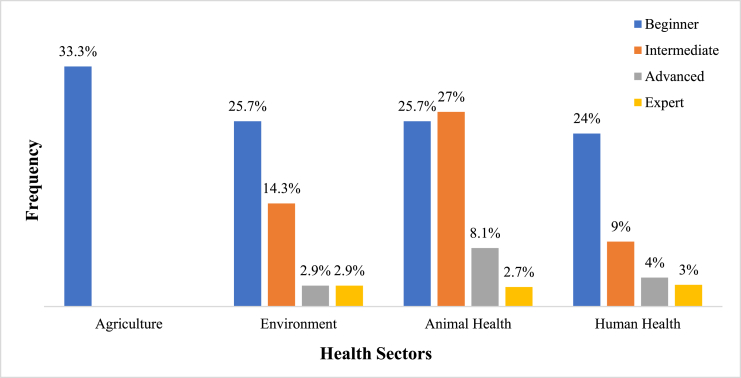
Assessment of the level of mastery of the One Health approach by professionals.

Up to half (57.6%) of the interviewed professionals had not received training that included the One Health concept. However, 42.4% of them had already received training including the One Health concept. They are mainly human resources in the human and animal health sectors. Various levels of mastery of the One Health concept were identified (Fig. 4): beginner (24.9%), intermediate (15.7%), advanced (5.1%), and expert (2.8%). Most professionals have a beginner to intermediate level, with a hegemony of those in the animal health sector. These competencies are obtained through training provided by Senegalese universities, national capacity building workshops organised by various international institutions, or even projects or continuing professional development outside of Senegal, in the context of cooperation or training grants.

**Fig. 5:**
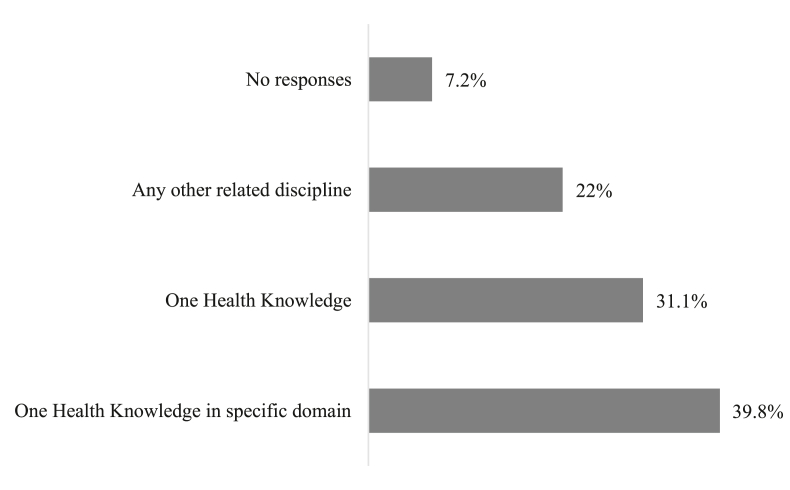
Areas to be strengthened in One Health training according to professionals.

**Fig. 6:**
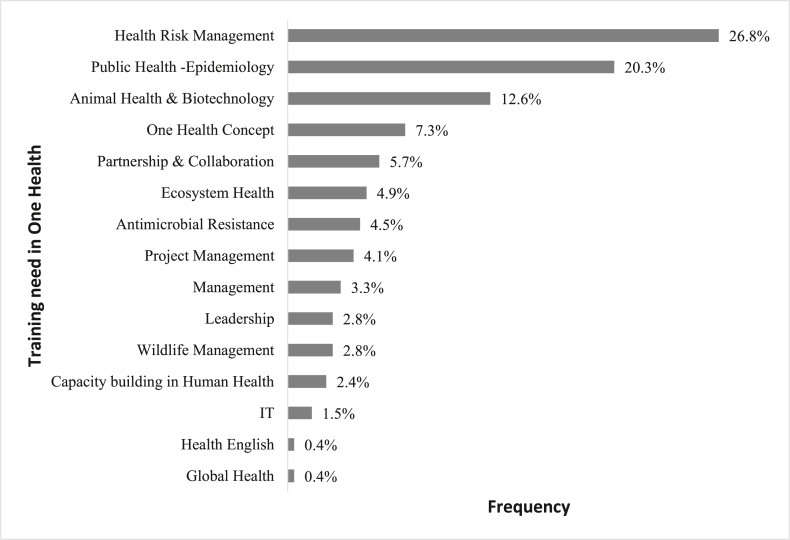
One Health training desired by professionals in Senegal.

Various training needs have been expressed by professionals (Fig. 5). Most of them (70.9%) wanted training focused on One Health knowledge or in a specific area of One Health. To this end (Fig. 6), interviewees mainly focused on Health Risk Management (30.4%) and everything related to public health and epidemiology (23%). These expressions of need intend to counterbalance the lack of knowledge about One Health or to progress on the level of mastery of this approach. The basic training received in the past by health professionals, with an average of 10 years of experience, only weakly integrated competencies in this approach.

The chi-square test reveals a significant difference with respect to the One Health domain for which training is desired by healthcare stakeholders (χ^2^ = 297.78, ddl = 15, 1-p = > 99.99%).

One Health competencies to be improved in professional career and/or in relation to the tasks related to their current positions vary according to the sector of activity (Table 3). The emphasis is more on technical skills than on soft skills. In the agriculture sector, interest is focused on One Health knowledge, while ecosystem health competency is more sought after in the fisheries sector. Skills related to health risk management and wildlife, mastering the One Health concept and animal health and biotechnology are required by environmental professionals. In the animal health sector, these are health risk management, public health and epidemiology and, to a lesser extent, collaboration and partnership, including the One Health concept. The discovery of animal health and biotechnology is a training need expressed by human health professionals, in addition to public health and epidemiology, management and health risk management. Antimicrobial resistance skills are more in demand among animal health and agriculture professionals than human health professionals.

**Table 3:** One Health competencies to be improved for professionals according to sector.

Competencies	Agriculture	Environment	Fishing	Animal Health	Human Health
Sanitary Risk Management	16.7%	22.9%		43.2%	10%
Wildlife Management		20.0%		1.4%	1%
Animal Health and Biotechnology		17.1%		9.5%	18%
Collaboration and Parternships	16.7%	2.9%		16.2%	3%
Management	16.7%	8.6%		4.1%	10%
One Health Concept	33.3%	20%		16.2%	6%
Public Health - Epidemiology	33.3%	11.4%		31.1%	17%
Leadership	16.7%	2.9%		5.4%	5%
Antimicrobial Resistance	16.7%			9.5%	2%
Ecosystemic Health		5.7%	100%	4.1%	8%
Public Health Capacity building					5%
IT		2.9%		1.4%	2%
Global Health					1%
Project Management					1%
Technico-Medical English					1%

## 4 Discussion

Epizootics (between 1987 and 2021) and epidemics (between 2012 and 2021) of Rift Valley fever observed in several regions of Senegal in animal transhumance corridors [[16], [17], [18], [19], [20]] could be quickly contained through to a collegial approach. The successes achieved have served to challenge the authorities on the role and impact of a holistic approach to effective intervention as described by the One Health approach.

In Senegal, the One Health approach was officially integrated into government action as a useful tool for managing health crises in 2016. A working platform on One Health has been set up at the highest level of the State to facilitate coordination and collaboration in health management between the actors involved [21,22]. After more than 5 years of existence, the reports of the evaluation missions highlight gaps in the implementation of the platform's activities, with exogenous funding coming exclusively from projects, the weak collaboration of stakeholders in information sharing and surveillance of priority diseases, etc. [21,23]. Weak collaboration may result from sector-oriented training of health professionals. However, training courses entirely dedicated to One Health are not available, for the moment, at universities in Senegal. Although teaching One Health is fundamental to the medical sciences [24], it is crucial to identify the right skills for the needs of each society. However, no study has yet been conducted in Senegal to identify and analyse the training needs of professionals in this approach. Such a study constitutes a real demand for the national One Health platform which wishes to offer agents training adapted (preparation, investigation, surveillance, response) to present and future needs in order to better deal with zoonoses, hence the justification for this study.

The One Health initiative, based on the close links between human, animal, and environmental health, has become a policy priority in designing disease prevention and control strategies and in ensuring preparedness for future pandemics [25]. Its implementation can help to effectively control and manage priority zoonotic diseases (Bovine Tuberculosis, Rabies, Highly Pathogenic Avian Influenza, Anthrax, Rift Valley Fever, Hemorrhagic disease Ebola virus and Marburg) in Senegal [26].

Rabies is one of the most notable examples of the application of One Health; however, recent studies show that animal health professionals handle better cases than human health professionals [[27], [28], [29], [30], [31], [32]], reflecting a weak level of collaboration between the two sectors. The lack of integrated management in infection control, particularly zoonotic ones, reflects the need to strengthen the capacities of professionals through harmonised training. Current good practice in rabies control involves a One Health approach, in which animal, human, and environmental health professionals work together through awareness campaigns and animal vaccination conducted by the Veterinary Services Department. This approach emphasizes collaborative efforts that harness and coordinate the power of multidisciplinary and cross-sectoral teams and resources to be applied at the local, national, and international levels for optimal human, animal, and environmental health [33]. The common theme in applying the One Health approach to rabies management is collaboration across disciplines and sectors.

According to Coulibando [34], the level of education is a country's human capital, the stock of knowledge and skills of its population. It is a measure of the wealth that education potentially confers on societies, economies and individuals. “The higher the educational level of the population, the better the prospects for the country's social, health, and economic progress” [35]. Thus, the professionals surveyed will have a sufficient level to execute, apply, and make decisions in their specific fields and in relation to the implementation of the One Health approach, in crisis and non-crisis situations, if these competencies are strengthened to manage emerging and re-emerging diseases with high-socioeconomic impact. The levels of One Health proficiency for trained professionals are concentrated at the beginner and intermediate levels. The “beginner” level in the agricultural sector must be linked to the approach initially focused on the three sectors of human, animal and environmental health. Beyond the level of professionals, the sustainability of the capacity building is a major issue [36] as there is a need to ensure continuity in the upgrading of agents to prepare them to respond to potential health issues, including disease outbreaks.

Several international initiatives (Asian, American, WHO, FAO, WHO) to promote the integration of the One Health approach in public health training have led to the definition of 7 key competency areas: management, communication and informatics, ethics and values, leadership, collaboration and partnership, roles and responsibilities, and systems thinking. However, as these competencies do not integrate the medical science domains, Togami et al. [12] proposed, after analyzing 45 public health training curricula from the United States, to combine antimicrobial resistance, zoonotic diseases, food safety, geographic information systems, emerging infectious diseases, epidemiology, plant biology, law, economics, toxicology, agriculture and livestock, politics, ecology and environmental health, vector-borne diseases, conservation and wildlife, and social and behavioural sciences. With leadership from the University of Minnesota, regional organisations such as One Health Central and Eastern Africa (OHCEA) and Southeast Asia One Health University Network (SEAOHUN) have identified and synthesized a set of One Health domains and competencies for use in the low-income countries context [11]. This study relied on the classification made by OHCEA; however, it does not define the levels of One Health mastery.

In Senegal, the interest of professionals appears to be focused on health risks, public health, epidemiology, and animal health, the most represented competencies in US institutions in the study by Togami et al. [12]. This particular interest in occupational health risk management is explained by the regularity of zoonotic disease outbreaks in West Africa since the advent of the Ebola Virus Disease in 2014. The COVID-19 global health context and the emergence and re-emergence of diseases such as Ebola, Highly Pathogenic Avian Influenza, and Rift Valley Fever are leading professionals to learn about the factors that lead to the emergence of these diseases and the management and response mechanisms that can be implemented to contain or even mitigate their societal effects. In addition, several training and response exercises in the framework of capacity building in terms of detection, prevention and control were organised for the human and animal health sectors. During the simulation exercises, in addition to health actors, administrative and territorial authorities as well as law enforcement agencies (police, customs, fire brigade, etc.) were invited. Following the importation of the first case of the Ebola Virus Disease in 2014 [37], human health actors benefited from training in Ebola response and intervention organised by the Health Emergency and Operations Centre (COUS) [38]. Similarly, in animal and environmental health, enhanced expertise following the 2018 HPAI H5N1 control and prevention simulation exercise [39] contributed to the rapid containment of HPAI H5N1 outbreaks in a poultry farm and in poultry in December 2020. This episode led to the closure of public and private reserves to contain the disease [40]. Following this first exercise, in January 2019, another simulation exercise on detection and response to a filovirus outbreak was organised at the initiative of the USAID PREDICT project. These courses are mainly aimed at professionals at level 8 according to the UNESCO classification (2013) [14]. As a result, lower level professionals lack opportunities to update their training if the offer is not supported by the public authority. Such a situation would render ineffective all collaborative efforts between sectors in the management of global health because of the role played by the latter category outside or in situations of health crises, regardless of the sector considered.

Managing these priority zoonotic diseases requires the activation of the usual response mechanisms of livestock, environmental, and public health services combined with collaborative actions. Joint activities between these three sectors, in addition to agriculture, result from the existence of coordinating bodies for the implementation of the One Health approach in Senegal (HCNSSM/OH^2^) and of training programs developed by the FAO called ISAVET (In Service Applied veterinary Epidemiology training [6]), and the WHO/CDC called FETP (Field Epidemiology Training Program [5]) over the last few years. The HCSSM/OH and the ISAVET and FETP trainings aim to improve the surveillance of transboundary and emerging diseases.

The aim of the training of human and veterinary professionals in field epidemiology is to fill the gap in specialist field epidemiologists identified in the PVS and JEE evaluations. ISAVET et FETP are basic (frontline) training courses aimed primarily at the doctorate, i.e. level 8 professionals (holders of a practising doctorate) according to the Unesco classification (2013) [14]. Its goal is to strengthen their field capacities to better manage emerging infectious diseases and transboundary animal diseases, and consequently to strengthen the national epidemiological surveillance and reporting system, based on sampling techniques, field diagnosis and consistent processing of the information generated. The aim is to anticipate, prepare for, respond to, and overcome critical emerging infectious disease threats at the interface of human, animal, and environmental health, and to raise awareness among affected communities of the essential measures to be implemented. To improve the performance of emerging disease preparedness and response systems, the FETP training programme on field epidemiology is designed for human health professionals.

Unlike ISAVET, this training goes beyond the basic level (frontline) and offers the intermediate level, delivered at the national level, and the advanced level delivered as a two-year Master's degree in Burkina Faso. These two courses have extremely low rates of access to other human and animal health professionals (veterinarians or doctors), which should be improved to facilitate the co-learning of agents who are called upon to pool their knowledge and competencies to effectively combat different types of public health threats.

Other existing courses around the world cover areas related to risk management and antibiotic resistance. The One Health: Connecting Humans, Animals and the Environment course at the University of Basel [41] and the Emerging Pandemic Threats (EPT) programme at USAID have solid experience in health risk management that would be an asset in building the capacity of professionals in Senegal. Through the PREDICT, PREVENT, IDENTIFY, RESPONSE projects, the EPT programme aims to strengthen the capacity of developing countries to prevent, detect and control infectious diseases in animals and humans, with an emphasis on early identification and response to dangerous animal pathogens before they become significant threats to human health [42]. The implementation of the activities of this programme has led to the training of some professionals and this would gain importance when integrated into a One Health training module.

In the face of the global scourge of antimicrobial resistance (AMR), it appears from our study that it is weakly solicited by professionals, along with other competencies such One Health concept, ecosystem health, wildlife management, collaboration and partnership, project management, health program management, leadership, etc. This finding is not specific to Senegal; it was also made by Togami et al. [12] in the United States. AMR is a global public health problem that, according to 2019 global estimates, will result in nearly 5 million human deaths associated with bacteria [43]. A global control plan has been agreed upon involving UN organisations such as WHO, WHOA, FAO to encourage action to mitigate antimicrobial use at regional and national levels [44]. The current state of knowledge shows that West Africa is the region most affected by AMR [43]. With the support of these organisations, NGOs and sub-regional projects, Senegal, through its One Health platform, has set up an AMR taskforce and has developed capacity building modules for students and professionals in the spirit of One Health. Fondation Mérieux has also developed a specific AMR module based on the One Health approach. It aims to combat AMR and build critical decision-making capacity in the developing world through education, partnerships and networks. It builds on the World Health Organization's (WHO) Global AMR Action Plan and the One Health global strategy, which identify awareness and understanding of AMR as a priority and essential for the adoption, deployment and implementation of national AMR action plans [45]. Antimicrobial resistance (AMR) resulting from antimicrobial use (AMU) is an emerging threat to global health. A key to better understanding and management of AMU and AMR is the development of effective and efficient integrated surveillance systems that take into account the complex epidemiology of these issues and the impacts of resistance on humans, animals and the environment [46,47]. In Senegal, Salmonella strains from poultry have been linked to human strains, highlighting concerns about the excessive use of antimicrobials on farms [48]. This highlights the need for active surveillance of salmonella and other common human and animal pathogens in the context of public health.

Gender is a key aspect of policy today [49,50]. While the trend towards more female students in medical fields is evident worldwide, particularly in Europe and Africa, the male gender predominates in health-related professions. This is also the case in Senegal, despite government efforts to keep girls in school and encourage their participation in scientific disciplines. Findings by Dieye [51] and Dahourou [52] revealed proportions ranging from 63% to 87% among boys and 13%–37% among girls in human and veterinary medical schools in Senegal. Gender equity has even been the subject of a national strategy. The low interest in gender competence by the health professionals surveyed could be related to its lack of impact on their career plans and/or core activities.

According to Togami et al. [12], without a One Health approach, environmental, animal, and human health experts will continue to address the challenges of health emergencies independently and uncoordinated. They will thus miss the opportunity to maximise the benefits of shared knowledge, common professional expertise, and available resources. One Health training has therefore the advantage of building the capacity of professionals to coordinate preparedness and response to public health emergencies and to address infectious disease threats in humans, animals and plants. As defined by Jennett and Laxdal [53], the gap between current and desired competencies inhibits optimal management of public health emergencies and can be addressed through tailored One Health training for all professionals.

The conduct (indirect via the internet and direct, i.e. face-to-face) and the context of the study mean that this work has some limitations. Firstly, Most health professionals work in the public sector, which is recognised as the largest employer in every country over the world. However, the sample adopted in this study does not allow for this comparison, as the public sector was primarily targeted. The three levels of human resource distribution (central, intermediate, and peripheral) are well represented in this study, as well as the multi-stakeholder aspect following the One Health approach. Secondly, the sample determined using the Thrusfield formula seems unrepresentative given the interest in the topic and the size of the target population. Although the representativeness of professionals by sector was respected, the small size of the sample due to the resources (logistical and financial) and the short time (12–22 September 2021) devoted to it gives the results obtained an exploratory character. Therefore, the analyses and conclusions of this study are at a more exploratory level. These various limitations need to be taken into account in the design and analysis of the data to build future studies.

## 5 Conclusion

Emerging and re-emerging diseases and antimicrobial resistance are challenging human resources in the human, animal and environmental health sectors in their mission to preserve global health. The present work is the first study in Senegal that assesses the training needs of professionals on One Health. The professionals had less than 10 years of experience and were predominantly male. The number of professionals with a relative level of decision-making was few. Slightly more than half of the professionals had not received training integrating the One Health approach. Those who received training in One Health acquired competencies at the beginner, intermediate and advanced levels; the expert level was absent. Senegalese universities and national and sub-regional projects such as the One Health Workforce have contributed to the development of these competencies. Nevertheless, most professionals (70.9%) expressed the need to learn more about One Health. Training needs are related to health risk management and public health and epidemiology. Professionals are more interested in technical competencies to acquire practical skills and attitudes to face the increasing health risks.

## Funding

This research is funded by USAID through the One Health Workforce-Next Generation (OHW-NG) Award 7200AA19CA00018, which is implemented by AFROHUN in Senegal. However, opinions and ideas developed in this paper are exclusively the responsibility of authors.

## Author contributions

Walter Ossebi, Andrée Prisca Ndjoug Ndour, Sara Danièle Dieng, André Pouwedeou Bedekelabou and Rianatou Bada-Alambedji: Conceptualization, Methodology. Walter Ossebi: investigation, leading original draft elaboration. Andrée Prisca Ndjoug Ndour Data curation, Formal analysis. Rianatou Bada-Alambedji: validation. Yalacé Yamba Kaboret, Adama Faye and Bienvenu Sambou: supervision, funding acquisition and strategic guidance. All authors: writing, review & editing.

## Conflict of interest

The authors declare that they have no conflict interests.

## Footnotes

1 https://www.who.int/news/item/01-12-2021-tripartite-and-unep-support-ohhlep-s-definition-of-one-health.

2 i.e. National high council for global health security.
